# Verbal autopsy analysis of childhood deaths in rural Gambia

**DOI:** 10.1371/journal.pone.0277377

**Published:** 2023-07-06

**Authors:** Baleng Mahama Wutor, Isaac Osei, Lobga Babila Galega, Esu Ezeani, Williams Adefila, Ilias Hossain, Golam Sarwar, Grant Mackenzie

**Affiliations:** 1 Medical Research Council Unit The Gambia at London School of Hygiene & Tropical Medicine, Fajara, The Gambia; 2 Faculty of Infectious and Tropical Diseases, London School of Hygiene & Tropical Medicine, London, United Kingdom; 3 Murdoch Children’s Research Institute, Melbourne, Australia; 4 Department of Paediatrics, University of Melbourne, Melbourne, Australia; Navrongo Health Research Centre, GHANA

## Abstract

**Background:**

In low-resource settings, it is challenging to ascertain the burden and causes of under-5 mortality as many deaths occur outside health facilities. We aimed to determine the causes of childhood deaths in rural Gambia using verbal autopsies (VA).

**Methodology:**

We used WHO VA questionnaires to conduct VAs for deaths under-5 years of age in the Basse and Fuladu West Health and Demographic Surveillance Systems (HDSS) in rural Gambia between September 01, 2019, and December 31, 2021. Using a standardized cause of death list, two physicians assigned causes of death and discordant diagnoses were resolved by consensus.

**Results:**

VAs were conducted for 89% (647/727) of deaths. Of these deaths, 49.5% (n = 319) occurred at home, 50.1% (n = 324) in females, and 32.3% (n = 209) in neonates. Acute respiratory infection including pneumonia (ARIP) (33.7%, n = 137) and diarrhoeal diseases (23.3%, n = 95) were the commonest primary causes of death in the post-neonatal period. In the neonatal period, unspecified perinatal causes of death (34.0%, n = 71) and deaths due to birth asphyxia (27.3%, n = 57) were the commonest causes of death. Severe malnutrition (28.6%, n = 185) was the commonest underlying cause of death. In the neonatal period, deaths due to birth asphyxia (p-value<0.001) and severe anaemia (p-value = 0.03) were more likely to occur at hospitals while unspecified perinatal deaths (p-value = 0.01) were more likely to occur at home. In the post-neonatal period, deaths due to ARIP (p-value = 0.04) and diarrhoeal disease (p-value = 0.001) were more likely to occur among children aged 1–11 months and 12–23 months respectively.

**Conclusion:**

According to VA analysis of deaths identified within two HDSS in rural Gambia, half of deaths amongst children under-5 in rural Gambia occur at home. ARIP and diarrhoea, and the underlying cause of severe malnutrition remain the predominant causes of child mortality. Improved health care and health-seeking behaviour may reduce childhood deaths in rural Gambia.

## Introduction

Reliable data on under-5 mortality is needed to effectively track progress towards attaining Sustainable Development Goal 3.2 which seeks to reduce neonatal and under-5 mortality to 12 and 25 per 1000 live births respectively by 2030 [1]. Globally, under-5 deaths in 2019 were estimated to be about 5.2 million [2]. More than half of these deaths occurred in Sub-Saharan Africa. Accurate mortality data is essential not only for planning and research purposes but also for measuring the impact of health interventions [3]. This is especially important in low and middle-income countries (LMICs) where resources are limited and need to be prioritized [4].

Although progress has been made in gathering data for evidence-based decision-making in child health, major gaps still exist [5]. Whereas death registration coverage is nearly 100% in the World Health Organisation (WHO) European Region, coverage is less than 10% in the African Region [6]. Even where Civil Registration and Vital Statistics (CRVS) systems exist in LMICs, they are often weak and incomplete [7]. Apart from the weak systems in these countries, a significant number of deaths in rural communities occur at home or health posts. Such deaths are not likely to be recorded and consequently, a cause of death will not be assigned. This paucity of mortality data tends to further perpetuate health inequalities [8].

Where Health and Demographic Surveillance Systems (HDSS) exist, they help fill this gap by ensuring that all deaths within those areas are captured [9]. This is especially useful in countries with weak CRVS systems and in areas where deaths occur at home and are not reported. However, to better understand the causes and circumstances within which these deaths occurred, the verbal autopsy (VA) tool was developed and refined over several years [6,10,11]. A VA tool is a structured questionnaire that is used to ascertain the cause of death by interviewing caregivers or the next of kin who were present during the illness and at the time of the death [6]. The caregiver or next of kin is asked about the signs and symptoms the deceased exhibited in the immediate days preceding their demise.

Verbal autopsy information is used to determine a cause of death using various methods. Some of these methods include physician-certified verbal autopsy (PCVA) [12] and computer-based probabilistic methods [13,14]. The widely used method of VA in sub-Saharan Africa is the PCVA which typically involves two physicians who assign a cause of death based on the information recorded in the VA interview [12,15]. Most validation studies have shown VA to be a reliable way to ascertain causes of death in the absence of robust CRVS systems [16]. Even though PCVA can be time-consuming and expensive to undertake, it remains a valuable tool for assigning causes for deaths occurring outside the healthcare setting [17]. Given its widespread usage, PCVA has been the most validated type of VA [15].

In this study, we sought to characterise causes of death assigned using PCVA within the Basse and Fuladu West HDSS sites in The Gambia.

## Methodology

### Study setting

The study was conducted in the Basse and Fuladu West HDSS sites located in the eastern part of The Gambia. The Gambia is a small country in West Africa with a population of about 2.4 million. According to the 2018 Gambia Multiple Indicator Cluster Survey, 15.4% of the population are children under-5 years of age and the mortality in this age group is about 57 per 1,000 live births [18].

**Fig 1:**
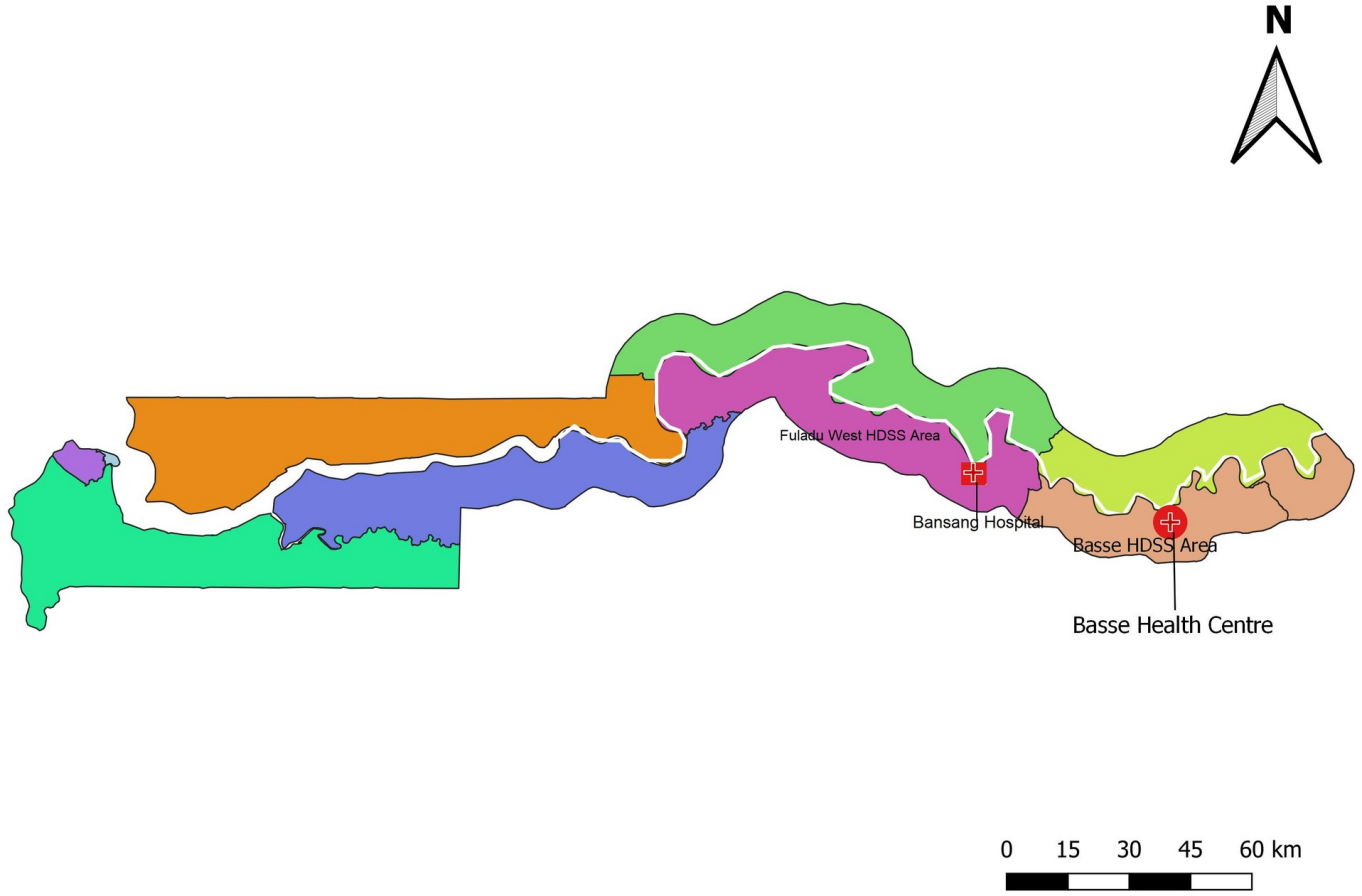
Map of The Gambia highlighting the Basse and Fuladu West HDSS sites.

In 2007, The Medical Research Council Unit The Gambia (MRCG) at the London School of Hygiene and Tropical Medicine (LSHTM) established the Basse HDSS in the Upper River Region of the Gambia (Fig 1). The Basse HDSS has been vital in the conduct of several large field studies [19–21]. It is located on the south bank of the River Gambia and covers an area of approximately 1,100 km^2^. This mostly rural area is inhabited by approximately 202,000 residents with 225 villages. Children aged less than 5 years make up 19% of the population. The Serahule and Fula tribes are the commonest ethnic groups in the area. The months from June to October experience a wet season, while the remaining months are dry.

The Fuladu West HDSS is located in the Central River Region and extends towards the west along the south bank of the River Gambia. It is made up of 213 villages with a total population of 99,113. The main hospital in the area is Bansang Hospital which receives referrals from the Central River Region as well as Upper River Region. The Basse and Fuladu West HDSSs currently support the cluster-randomized Pneumococcal Vaccine Schedules clinical trial [22].

### Study design

This cross-sectional observational study was conducted between September 01, 2019, and December 31, 2021. All deaths of children under-5 who were residents of any of the two HDSSs were eligible for inclusion in the study. Residency was defined as living within the HDSSs in the last 4 months as confirmed by HDSS records. Verbal autopsies were conducted for 647 of 727 (89%) detected deaths of children aged less than 5 years during the observation period. 80 VAs could not be conducted because the caregivers who were present during the period of illness had migrated out of the HDSS sites and could not be traced.

### Procedure

The VAs were coded by two pairs of physicians, all of whom were active in paediatric care in the two main referral hospitals in the study area (Basse and Bansang). They were specifically trained on how to code deaths using the PCVA method as part of safety surveillance for the Pneumococcal Vaccine Schedule study [22]. Each physician worked independently to assign a probable cause of death based on the information captured in the VA using their clinical judgement. In each case, a primary and underlying cause of death was coded. The disease condition or terminal event which occurred just before the death was selected as the primary cause of death. The morbid condition or injury which initiated the chain of events leading to the death was selected as the underlying cause of death [23].

Where there was a single cause of death, it was considered both the underlying and primary cause of death. The diagnosis of severe malnutrition was restricted to children between the ages of 1–59 months. If a baby was born showing signs of life but later died within the first 27 days of life, this was considered a neonatal death. Children born without any signs of life (as confirmed by several questions in the VA questionnaire) were classified as still births. For neonatal VA forms where a specific cause of death could not be assigned after reviewing all the signs and symptoms or where there was insufficient information in the VA form, the cause of death assigned was, unspecified perinatal cause of death. This approach is similar to other VA studies [24,25].

After coding, the results of both physicians were compared, and discrepancies were resolved by consensus among the two physicians. Causes of death were assigned using a shortlist of deaths (S2 File) that could be ascertained from VA and mapped to the International Classification of Disease 10 codes. This list is contained in the 2016 WHO VA instrument [26].

### Data collection

Trained field workers undertake household visits within the Basse and Fuladu West HDSSs every 4 months. Data on all deaths, births, migrations, and pregnancies, are captured during visits. Verbal autopsies are conducted routinely after a grieving period of 40 days. The interviews are conducted using standardised closed-ended WHO VA questionnaires designed for neonates and children 1–59 months of age [27]. Field workers were trained on each item of the questionnaire and how to conduct interviews with respect and understanding. During interviews, the questions are translated by field workers from English into the appropriate local language of the respondent. Corresponding data is captured in a tablet application using the Research Electronic Data Capture (REDCap) application database system [28].

### Statistical analysis

All analyses were performed using Stata version 17 (StataCorp, College Station, TX, USA). Data were assessed for errors and missing values. Age was categorized into five groups: stillbriths, neonates (0–27 days), 1–11 months, 12–23 months, and 24–59 months. Variables with few numbers were grouped as “other”. Summary statistics were performed for key characteristics such as socio-demographic factors, primary causes of death, underlying causes of death, and contextual factors (decision to seek care, access to medical care, and adequacy of care). The summary of the results is presented using frequency tables and percentages. Bivariate analysis using Chi-square test or the Fisher’s exact test was performed to determine possible association between some key socio-demographic factors and the top five neonatal and post-neonatal causes of death. We determined statistical significance at a p-value of ≤ 0.05.

### Ethical consideration

The collection of VAs was undertaken by the Basse and Fuladu West HDSSs as part of safety surveillance in the Pneumococcal Vaccine Schedules trial. [22] The HDSSs include hundreds of thousands of residents and constitute minimal-risk observational research with data used by multiple research projects, non-government organisations, and government agencies. For these reasons, verbal consent was sought from the head of each household at each household visit and VA. The operation and procedures of both the Basse and Fuladu West HDSSs were approved by the Gambia Government/MRCG Joint Ethics Committee (reference: 1577) and the London School of Hygiene and Tropical Medicine Ethics Committee (reference: 14515).

## Results

### Socio-demographic characteristics

Table 1 describes the socio-demographic characteristics of the evaluated deaths. Most of the deaths occurred in the neonatal period (32.3%, n = 209). Outside of the neonatal period, 175 (27.0%) deaths occurred in the first year of life, 89 (13.8%) in the second year of life, and 143 (22.1%) occurred in children aged 24–59 months (22.1%, n = 143). There were 31 (4.8%) stillbirths. There were comparable numbers of male and female deaths, with 324 (50.1%) females and 321 (49.6%) males. Most of the deaths occurred amongst the Serahule tribe (34.6%, n = 224) and the Fula tribe (32.2%, n = 208) which represent 32.5% and 39.3% of the under-5 years population in the study area. Almost all the respondents who gave information relating to the circumstances of the deaths were family members, with 92.9% (n = 601) being the parent(s) of the deceased. During the period of illness that led to death, 97.5% (n = 631) of the respondents lived with the deceased. Concerning seasonality, 39.9% (n = 258) of the deaths occurred during the 5 months of the wet season. The majority of deaths occurred at home (49.5%, n = 319).

**Table 1:** Socio-demographic characteristics of the deceased.

Variables	Frequency (n = 647) *	Percentage (%)
**Age group**		
Stillbirths	31	4.8
Neonates (0-27days)	209	32.3
1-11months	175	27.0
12–23 months	89	13.8
24–59 months	143	22.1
**Sex**		
Female	324	50.1
Male	321	49.6
**Ethnicity**		
Fula	208	32.2
Serahule	224	34.6
Wolof	42	6.5
Mandinka	162	25.0
Others	11	1.7
**Relationship of respondent to the deceased**		
Parent	601	92.9
Family member	43	6.7
Another relationship	3	0.5
**Did respondent live with the deceased during the illness?**		
Yes	631	97.5
**Season of death**		
Wet	258	39.9
Dry	389	60.1
**Place of death**		
Home	319	49.5
Hospital	179	27.8
Other health facility	123	19.1
En route to the hospital	17	2.6
Other	6	0.9

*Due to missing values, not all N values equal 647; sex = 2 missing values, Did the respondent live with the deceased = 7 missing values, Place of death = 3 missing values.

### Contextual factors

In the final days of the illness, most parents knew that the deceased needed medical care (72.2%, n = 464). However, care was not sought outside the home in 63.7% (n = 412). For those who sought care outside the home, most (90.9%, n = 210) went to a health facility (health centre and hospital). A few respondents used a mobile phone to call for help during the period of illness (12.3%, n = 79). In most cases, the deceased was sent to a health facility in the final days of the linness (71.3%, n = 460). Only few respondents reported living more than 2hrs journey from a health facility (11.8%, n = 76). In 72.9% (n = 471) of cases, the deceased received some form of treatment during the illness. For those who visited a health facility, only a few (1.7%, n = 8) of respondents reported having problems with how the deceased were treated (treatment, procedures, attitude, respect, dignity) in the hospital/health facility. A few respondents reported having challenges getting medications or diagnostic tests at health facilities (12.8%, n = 59) (Table 2).

**Table 2:** Contextual factors associated with deaths.

Contextual factor	Frequency (n = 647) *	Percentage (%)

**1. Decision to seek care**		
**In the final days of illness, were there any doubts whether medical care was needed?**		
No	464	72.2
**Sought care outside home**		
No	412	63.7
**Where was care sought?**		
Traditional Healer	7	3.0
Hospital	57	24.7
Health Centre	153	66.2
Other	14	6.1
**In the final days of illness, did anyone use a phone to call for help?**		
Yes	79	12.3
**2. Access to medical care**		
**Does it take more than 2hrs to get to the nearest hospital/health facility**		
Yes	76	11.8
^¥^ **In the final days before death, did s/he travel to a hospital or health facility?**		
Yes	460	71.3
**3. Adequacy of medical care**		
**Did (s)he receive any treatment for the illness that led to death?**		
Yes	471	72.9
**Were there any problems with the way (s)he was treated in the hospital or health facility** †		
Yes	8	1.7
**Were there any problems getting medications or diagnostic tests in the hospital or health**† **facility?**		
Yes	59	12.8

* Due to missing data, not all N categories sum up to 647.

Missing values: In the final days of illness, were there any doubts whether medical care was needed? = 4 missing values, Sought care outside home = 4 missing values, In the final days of illness, did anyone use a phone to call for help? = 2 missing values, Does it take more than 2hrs to get to the nearest hospital/health facility = 2 missing values, In the final days before death, did s/he travel to a hospital or health facility? = 2 missing values, Did (s)he receive any treatment for the illness that led to death? = 1 missing value, Were there any problems with the way (s)he was treated in the hospital or health facility = 1 missing value.

^¥^ Question indicates whether the deceased had any contact with a health facility.

^†^ Total frequency = 462.

### Primary causes of death

Of all deaths, acute respiratory infection including pneumonia (ARIP) (21.2%, n = 137), diarrhoeal diseases (14.7%, n = 95), sepsis (13.6%, n = 88) and unspecified perinatal causes of death (11.0%, n = 71) were the commonest primary causes of death (Table 3). S1 Table in the shows a further breakdown of causes of death in the neonatal and post-neonatal periods of life. In the neonatal period, unspecified perinatal causes of death (34.0%, n = 71), birth asphyxia (27.3%, n = 57) and prematurity/low birth weight (19.6%, n = 41) were the commonest causes of death. Outside the neonatal period, ARIP (33.7%, n = 137), diarrhoeal diseases (23.3%, n = 95) and sepsis (21.6%, n = 88) were the commonest primary causes of death. Of note, there were few malaria deaths (0.8%, n = 5).

**Table 3:** Primary causes of death.

Primary cause of death	Frequency (N = 647)	Percentage (%)
All deaths		
Acute respiratory infection including Pneumonia (ARIP)	137	21.2
Diarrhoeal diseases	95	14.7
Sepsis	88	13.6
Unspecified perinatal cause of death	71	11.0
Birth asphyxia	57	8.8
Prematurity/Low birth weight	41	6.3
Fresh stillbirth	30	4.6
Meningitis	26	4.0
Severe anaemia	26	4.0
Cause of death unknown	24	3.7
Neonatal Sepsis	16	2.5
Road traffic accident	6	0.9
Malaria	5	0.8
Neonatal pneumonia	4	0.6
Others	21	3.2

### Underlying causes of death

Severe malnutrition (28.6%, n = 185), ARIP (14.4%, n = 93), unspecified perinatal deaths (10.7%, n = 69), and prematurity/low birth weight (10.2%, n = 66) were the commonest underlying causes of death (Table 4). S2 Table in the shows a further breakdown of the underlying causes of death in the neonatal and post-neonatal periods of life. Of the neonatal deaths that were coded, 69 (30.0%) had an underlying cause recorded as unspecified perinatal neonatal deaths, 60 (26.1%) were associated with prematurity/low birth weight, and the underlying cause of death was unknown in 32 (13.9%) cases. Among the deaths which occurred outside of the neonatal period, severe malnutrition (45.2%, n = 184), ARIP (22.9%, n = 93), and sepsis (15.7%, n = 64) were the top three underlying causes of death.

**Table 4:** Underlying causes of death.

Underlying causes of death	Frequency (N = 647)	Percentage (%)
All deaths		
Severe malnutrition	185	28.6
Acute respiratory infection (including Pneumonia)	93	14.4
Unspecified perinatal cause of death	69	10.7
Prematurity/Low birth weight	66	10.2
Sepsis	64	9.9
Cause of death unknown	42	6.5
Fresh stillbirth	29	4.5
Birth Asphyxia	28	4.3
Neonatal Sepsis	13	2.0
Diarrhoeal diseases	8	1.2
Malaria	8	1.2
Meningitis	8	1.2
Neonatal Pneumonia	6	0.9
Road traffic accident	6	0.9
Accidental fall	3	0.5
Contact with venomous animals and plants	3	0.5
Others	16	2.5

### Factors associated with under-5 deaths

In the neonatal period, we did not find any significant association between sex and the top five primary causes of death (Table 5). While deaths from birth asphyxia (p-value<0.001) and Severe Anaemia (p = 0.03) were more likely to occur at hospitals, unspecified perinatal deaths were more likely to occur at home (p = 0.01). Deaths associated with prematurity and low birth weight were more likely to occur at other health facilities (p = 0.04) and in the wet season (p = 0.03).

**Table 5:** Factors associated with neonatal deaths.

	Top five neonatal primary causes of death									
Variable	Unspecified perinatal deaths		Birth asphyxia		Prematurity and Low birth weight		Neonatal Sepsis		Severe Anaemia	
	n (%)	p-value	n (%)	p-value	n (%)	p-value	n (%)	p-value	n (%)	p-value
**Sex**										
*Female*	34(35.8)	0.72	28(29.5)	0.77	19(20.0)	0.87	4(4.2)	0.63	2(1.5)	0.31
*Male*	35(33.3)		29(27.6)		22(20.9)		6(5.7)		5(4.8)	
**Place of death**										
*Hospital*	17(24.6)	0.01	33(47.8)	<0.001	7(10.1)	0.04	2(2.9)	0.36	6(8.7)	0.03
*Home*	39(46.4)		12(14.3)		19(22.6)		4(4.8)		0(0.0)	
*Other health facility*	10(25.0)		11(27.5)		13(32.5)		4(10.0)		1(1.19)	
**Season**										
*Wet*	24(31.2)	0.43	19(24.7)	0.34	22(28.6)	0.03	2(2.6)	0.22	4(5.2)	0.30
*Dry*	45(36.6)		38(30.9)		19(15.5)		8(6.5)		3(2.4)	

In the post-neonatal period, age was statistically associated with deaths from ARIP, diarrhoeal diseases, and sepsis (Table 6). Those aged 1–11 months were more likely to die from ARIP (p-value = 0.04) and sepsis (p-value = 0.05), while those aged 12–23 months were more likely to die from diarrhoeal diseases (p-value = 0.001). Sex, place of death and season were not found to be associated with the top five post-neonatal causes of death.

**Table 6:** Factors associated with post-neonatal primary causes of death.

	Top five post-neonatal primary causes of death									
Variable	ARIP		Diarrhoeal diseases		Sepsis		Meningitis		Severe Anaemia	
	n (%)	p-value	n (%)	p-value	n (%)	p-value	n (%)	p-value	n (%)	p-value
**Age group**										
*1–11 months*	71(39.0)	0.04	28(15.5)	0.001	47(26.0)	0.05	9(4.9)	0.24	6(3.3)	0.54
*12-23months*	22(25.0)		31(35.2)		12(13.6)		4(4.5)		5(5.7)	
*24-59months*	43(29.9)		36(25.0)		28(19.4)		13(9.0)		8(5.6)	
**Sex**										
*Female*	79(36.2)	0.09	49(22.5)	0.85	49(22.5)	0.41	11(5.0)	0.28	12(5.5)	0.22
*Male*	57(28.6)		46(23.2)		38(19.2)		15(7.6)		6(3.0)	
**Place of death**										
*Hospital*	36(35.3)	0.27	21(20.6)	0.62	23(22.6)	0.86	7(6.9)	0.91	6(5.9)	0.95
*Home*	71(30.7)		55(23.9)		49(21.3)		15(6.5)		10(4.3)	
*Other health facility*	21(31.3)		18(26.9)		13(19.5)		3(4.5)		3(4.5)	
**Season**										
*Wet*	55(32.7)	0.94	41(24.4)	0.52	41(24.4)	0.14	10(5.9)	0.84	4(2.4)	0.08
*Dry*	81(32.4)		54(21.7)		46(18.5)		16(6.4)		15(6.0)	

## Discussion

In this verbal autopsy analysis of deaths of children under-5 years of age in rural Gambia, we found that half of all deaths occurred at home. ARIP, diarrhoeal diseases, and sepsis were the leading primary causes of death. Severe malnutrition contributed to nearly half of the post-neonatal deaths. The highest number of deaths was in the neonatal period with the commonest causes of death being unspecified perinatal deaths, birth asphyxia, and prematurity/low birth weight. We also found that though most of the respondents were aware that the deceased needed medical care and lived within 2hrs of a health facility, care was not sought outside the home in most cases.

One reason children may be dying at home is the reluctance of parents or caretakers to take them to the hospital when they are sick. This could be because they did not think the sickness was serious or they resorted to treatment at home. Another reason could be if treatment was sought in places other than health facilities. However, in this study, only a few caretakers reported seeking care from traditional healers or pharmacies. Other studies in Ghana, Rwanda and Ethiopia found a similarly high number of home deaths [29–31]. With substantial proportions of deaths occurring at home, mortality data generated through the use of VA should be considered as an adjunct to data from health facilities in characterizing under-5 deaths.

We also found that almost a third of deaths occurred in the neonatal period (32.3%). The high burden of deaths in the neonatal period was equally noted in an HDSS site in the northern part of India [32]. A significant proportion of births in The Gambia still occur without the supervision of skilled birth attendants and may be a contributing factor to the unacceptably high number of neonatal deaths [18]. From our experience, such neonates are often brought to the health facility a day or two after developing complications such as jaundice or sepsis.

Severe malnutrition and ARIP were the commonest underlying and primary causes of death respectively. This is similar to verbal autopsy studies in Ethiopia and Bangladesh where acute lower respiratory infection was the commonest cause of death [30,33]. In the latter study, childhood wasting was additionally identified as the leading risk factor for lower respiratory infections. This is corroborated by case-control studies from The Gambia and Brazil which identified malnutrition as the most important risk factor for pneumonia in children [34,35]. These findings highlight the synergistic role that acute malnutrition and respiratory infections play in causing the demise of many children. Apart from malnutrition, another risk factor that may have contributed to the high number of ARIP deaths is indoor pollution from the use of charcoal and firewood for cooking. This has been identified as a major risk factor contributing to pneumonia deaths in The Gambia [36–38]. Therefore, measures aimed at reducing ARIP deaths should also consider addressing malnutrition and indoor pollution from smoke.

Unspecified perinatal neonatal death was the commonest cause of death in the neonatal period. This is the case because of the challenge in assigning specific causes of death using verbal autopsy for neonates who die in the first few days of life. This challenge was equally recognised by Baqui et al., who in a nationwide verbal autopsy analysis grouped neonatal deaths in the first three days of life as early perinatal deaths without further specifying [33]. A similar verbal autopsy study in Angola also identified unspecified neonatal deaths as the commonest cause of death in the neonatal period [25]. In this study, a contributing factor could have been some skip pattern errors in some of the neonatal VA forms which resulted in some missing values. This further compounded the difficulty in assigning a specific cause of death in such cases.

One striking finding in this study was the low number of deaths attributable to malaria. In the past, malaria was a common cause of death in The Gambia but its incidence has fallen dramatically in recent years [39,40]. The current low prevalence of malaria in The Gambia is a result of multiple malaria control interventions, such as the use of insecticide-treated bed nets, seasonal malaria chemoprophylaxis, effective treatment and community education [39–41].

The social circumstances that contributed to the deaths were grouped using the three delays model (decision to seek care, access to care, and adequacy of care) [42]. Even though most of the caretakers did not doubt that the deceased needed medical care at the time of the illness, a significant proportion did not seek care outside of the home. One reason could have been the issue of access to health facilities. However, the overwhelming majority reported that it did not take more than 2hrs to get to the nearest health facility. A second reason for the reluctance in seeking medical care could have been the (perceived) quality of care that children receive at health facilities. However, most of the respondents reported no problems in the way the children were treated, and in obtaining medications and other diagnostic tests. This may have been due to response bias.

Significant associations were noted between neonatal deaths and place of death. Deaths from birth asphyxia and severe anemia were more likely to occur at health facilities whereas unspecified perinatal deaths were more likely to occur at home. Deaths from birth asphyxia may be occurring more at health facilities because women who had complicated pregnancies or deliveries were likely to report to health facilities. However, late recognition of danger signs, lack of access to health facilities or delays in referral systems may have resulted in them receiving medical care too late, hence, giving birth to asphyxiated babies. More unspecified perinatal deaths may have been reported from home because of the inability of mothers to fully describe the signs and symptoms related to such deaths. Compared to children who die at home, those who die at health facilities are likely to be told the specific cause of death and the important symptoms by health workers.

In the post-neonatal period, ARIP and sepsis deaths were likely to occur in children aged 1–11 months whiles diarrhoeal deaths were likely to occur in children aged 12–23 months. Compared to older children, infants have a less resilient immune system and so are more likely to succumb to infections like ARIP. Also, diarrhoeal deaths may have been more in older children because the latter being more active and increasingly independent are more likely to consume contaminated food and water compared to younger children who are still fully dependent on their parents.

### Strengths/Limitations

A strength of our study is using WHO’s standardised VA questionnaire (S3 File) and cause of death list (S2 File). The advantage of this is that it allows our results to be compared to similar studies conducted using the same questionnaire. The relatively large sample size in this study also strengthens the conclusions drawn. Another strength is that most of the respondents were first-degree relatives of the deceased, so more accurate information was likely to be collected.

However, VA analyses have some inherent limitations. Translation of the VA questionnaire from English to the local languages may have caused some meaning to be lost or misinterpreted. To mitigate this, only field workers who were fluent in the language and understood the culture and customs of the respondent were allowed to conduct such VAs. Possible recall bias is another well-known limitation of VA. To mitigate this in our study, we ensured that VAs were conducted as soon as possible, being mindful however of the need for relatives to mourn the deceased. It is also possible that some deaths remain unreported, despite HDSS procedures.

Validation studies comparing VA assigned causes of death to hospital records have shown mixed results [15,16,43]. Therefore, though deaths assigned using VA are important in settings with weak systems for registering and determing causes of death, they should be interpreted with caution.

## Conclusions

Verbal autopsies are useful in assigning likely causes of child deaths in settings with weak CRVS systems. Half of the deaths amongst children aged under-5 in rural Gambia occur at home. ARIP and severe malnutrition are the commonest primary and underlying causes of death respectively. Though most respondents were aware of the need for medical care and lived close to health facilities, a significant proportion did not seek medical care.

We recommend the integration of VA methods in the national CRVS to ensure complete and accurate data on under-5 deaths. Improved health care and health-seeking behaviours through health education and promotion could contribute to reducing the number of under-5 deaths in rural Gambia. This, however, should be part of a comprehensive plan to improve health care at all levels including more trained personnel and improved logistics at health facilities.

## Supporting information

S1 Table Primary causes of death. (DOCX)

S2 Table Underlying causes of death. (DOCX)

S1 File Minimal data set V 1.0. (DTA)

S2 File VA Coding list_V1.0.pdf. (PDF)

S3 File WHO VA questionnaire. (XLSX)
