# Supplementary material for: Verbal autopsy analysis of childhood deaths in rural Gambia
Source: PLoS One. 2023 Jul 6;18(7):e0277377. doi: 10.1371/journal.pone.0277377 (PMC10325104; doi:10.1371/journal.pone.0277377)
Supplement: S1 Table — (DOCX) [file pone.0277377.s001.docx]

**S1 Table. Primary causes of death.**

| **Primary cause of death** | **Frequency (N=647)** | **Percentage (%)** |
| --- | --- | --- |
| **All deaths** | | |
| Acute respiratory infection including Pneumonia (ARIP) | 137 | 21.2 |
| Diarrhoeal diseases | 95 | 14.7 |
| Sepsis | 88 | 13.6 |
| Unspecified perinatal cause of death | 71 | 11.0 |
| Birth asphyxia | 57 | 8.8 |
| Prematurity/Low birth weight | 41 | 6.3 |
| Fresh stillbirth | 30 | 4.6 |
| Meningitis | 26 | 4.0 |
| Severe anaemia | 26 | 4.0 |
| Cause of death unknown | 24 | 3.7 |
| Neonatal Sepsis | 16 | 2.5 |
| Road traffic accident | 6 | 0.9 |
| Malaria | 5 | 0.8 |
| Neonatal pneumonia | 4 | 0.6 |
| Others | 21 | 3.2 |
| **Neonatal period (0-27days)** | | |
| Unspecified perinatal cause of death | 71 | 34.0 |
| Birth asphyxia | 57 | 27.3 |
| Prematurity/ Low birth weight | 41 | 19.6 |
| Neonatal sepsis | 16 | 7.7 |
| Cause of death unknown | 13 | 6.2 |
| Severe anaemia | 6 | 2.9 |
| Neonatal pneumonia | 4 | 1.9 |
| Accidental fall | 1 | 0.5 |
| **1month-59months** | | |
| Acute respiratory infection including Pneumonia (ARIP) | 137 | 33.7 |
| Diarrhoeal diseases | 95 | 23.3 |
| Sepsis | 88 | 21.6 |
| Meningitis | 26 | 6.4 |
| Severe Anaemia | 20 | 4.9 |
| Cause of death unknown | 11 | 2.7 |
| Road traffic accident | 6 | 1.5 |
| Malaria | 5 | 1.2 |
| Severe malnutrition | 4 | 1.0 |
| Contact with venomous animals and plants | 3 | 0.7 |
| Accidental exposure to smoke fire and flames | 2 | 0.5 |
| Assault | 2 | 0.5 |
| Unspecified cardiac disease | 2 | 0.5 |
| Accidental drowning and submersion | 1 | 0.3 |
| Others | 5 | 1.2 |
