# Supplementary material for: Verbal autopsy analysis of childhood deaths in rural Gambia
Source: PLoS One. 2023 Jul 6;18(7):e0277377. doi: 10.1371/journal.pone.0277377 (PMC10325104; doi:10.1371/journal.pone.0277377)
Supplement: S2 Table — (DOCX) [file pone.0277377.s002.docx]

**S2 Table. Underlying causes of death.**

| **Underlying causes of death** | **Frequency (N=647)** | **Percentage (%)** |
| --- | --- | --- |
| **All deaths** | | |
| Severe malnutrition | 185 | 28.6 |
| Acute respiratory infection including Pneumonia (ARIP) | 93 | 14.4 |
| Unspecified perinatal cause of death | 69 | 10.7 |
| Prematurity/Low birth weight | 66 | 10.2 |
| Sepsis | 64 | 9.9 |
| Cause of death unknown | 42 | 6.5 |
| Fresh stillbirth | 29 | 4.5 |
| Birth Asphyxia | 28 | 4.3 |
| Neonatal Sepsis | 13 | 2.0 |
| Diarrhoeal diseases | 8 | 1.2 |
| Malaria | 8 | 1.2 |
| Meningitis | 8 | 1.2 |
| Neonatal Pneumonia | 6 | 0.9 |
| Road traffic accident | 6 | 0.9 |
| Accidental fall | 3 | 0.5 |
| Contact with venomous animals and plants | 3 | 0.5 |
| Others | 16 | 2.5 |
| **Neonatal (0days-27days)** | | |
| Unspecified perinatal cause of death | 69 | 30.0 |
| Prematurity/Low birth weight | 60 | 26.1 |
| Cause of death unknown | 32 | 13.9 |
| Birth asphyxia | 28 | 12.2 |
| Neonatal sepsis | 11 | 4.8 |
| Neonatal pneumonia | 6 | 2.6 |
| Accidental fall | 1 | 0.4 |
| Congenital malformation | 1 | 0.4 |
| Others | 2 | 0.8 |
| **1month-59 months** | | |
| Severe malnutrition | 184 | 45.2 |
| Acute respiratory infection (including pneumonia) | 93 | 22.9 |
| Sepsis | 64 | 15.7 |
| Cause of death unknown | 10 | 2.4 |
| Diarrhoeal diseases | 8 | 2.0 |
| Malaria | 8 | 2.0 |
| Meningitis | 8 | 2.0 |
| Prematurity/Low birth weight | 6 | 1.5 |
| Road Traffic Accident | 6 | 1.5 |
| Contact with venomous animals and plants | 3 | 0.7 |
| Accidental exposure to smoke fire and flames | 2 | 0.5 |
| Accidental fall | 2 | 0.5 |
| Assault | 2 | 0.5 |
| Others | 11 | 2.7 |
