# Supplementary material for: Verbal autopsy analysis of childhood deaths in rural Gambia
Source: PLoS One. 2023 Jul 6;18(7):e0277377. doi: 10.1371/journal.pone.0277377 (PMC10325104; doi:10.1371/journal.pone.0277377)
Supplement: S2 File — (PDF) [file pone.0277377.s004.pdf]

VA code	Verbal Autopsy title	ICD 10 code
01.01	Sepsis	A41
01.02	Acute respiratory infection including pneumonia	J22, (J00-J22), J85
01.03	HIV/AIDS related death	B24
01.04	Diarrheal diseases	A09
01.05	Malaria	B54
01.06	Measles	B05
01.07	Meningitis	G03
01.07	Encephalitis	G04
01.08	Tetanus	A35
01.09	Pulmonary Tuberculosis	A16
01.10	Pertussis	A37
01.11	Haemorrhagic fever	A99
01.99	Other unspecified infectious diseases	B99
02.99	Other unspecified Neoplasms	C80
03.01	Severe Anaemia	D64
03.02	Severe Malnutrition	E46
03.03	Diabetes Mellitus	E14
04.03	Sickle Cell disease	D57
04.99	Other and unspecified cardiac disease	I99
05.01	Chronic Obstructive Respiratory disease (COPD)	J44
05.02	Asthma	J45
06.01	Acute abdomen	R10

06.02	Liver cirrhosis	K74
07.01	Renal Failure	N19
08.01	Epilepsy	G40
10.01	Prematurity or Low Birth weight	P07
10.02	Birth Asphyxia	P21
10.03	Neonatal Pneumonia	P23
10.04	Neonatal Sepsis	P36
10.05	Neonatal tetanus	A33
10.06	Congenital Malformation	Q89
10.99	Other and unspecified perinatal cause of death	P96
11.01	Fresh stillbirth	P95
11.02	Macerated stillbirth	P95
12.01	Road Traffic Accident	V89
12.02	Other transport accident	V99
12.03	Accidental fall	W19
12.04	Accidental drowning and submersion	W74
12.05	Accidental exposure to smoke, fire, and flames	X09
12.06	Contact with venomous animals and plants	X29
12.07	Accidental poisoning and exposure to noxious substance	X49
12.08	Intentional self-harm	X84
12.09	Assault	Y09
12.10	Exposure to force of nature	X39
12.99	Other and unspecified external cause of death	X59
99.99	Cause of death unknown	R99
